# Return to sport assessment following isolated anterior cruciate ligament reconstruction - 82 consecutive cases

**DOI:** 10.3389/fspor.2026.1922271

**Published:** 2026-09-30

**Authors:** Suchung Kim, Michael Cassel, Lucca Lacheta, Marc Trefz, Ulrich Stoeckle, Oliver Miltner

**Affiliations:** 1 Center of Musculoskeletal Surgery, Charité Medical University Hospital Berlin, Augustenburgerplatz, Berlin, Germany; 2 Ortho Eins, private outpatient clinic for orthopedic and sports medicine, Berlin, Germany; 3 Sports Medicine and Sports Orthopaedics, University Outpatient Clinic, University of Potsdam, Potsdam, Germany; 4 Sektion Sportorthopädie, TUM Klinikum Rechts der Isar, Ismaninger Straße, Munich, Germany; 5 Praxis TWELVE, Dr. med. Marc Trefz, Possartstraße, Munich, Germany; 6 DocOrtho MVZ GmbH, Friedrichstraße, Berlin, Germany

**Keywords:** anterior cruciate ligament reconstruction, hop test, isokinetic strength, outpatient clinic, rehabilitation, return to sport

## Abstract

**Purpose:**

The purpose of this study was to investigate different patient related factors associa/copted with objective return to sport (RTS) testing after an isolated anterior cruciate ligament reconstruction (ACLR) following an optimised and standardized outpatient clinic setting.

**Methods:**

From 2015 to 2019, patients with isolated ACL tears undergoing ACL reconstruction (ACLR) with hamstring autograft were included. All followed the same standardized rehabilitation protocol. RTS testing comprised isokinetic extensor (ISO Ext) and flexor (ISO Flex) strength, One-Leg Hop (OLH), Side Hop (SH), and Tapping Agility (TT). A Limb Symmetry Index (LSI) ≥ 90% defined the pass criterion for strength and hop tests; H/Q ≥ 50% was used for both limbs. Logistic regression assessed the influence of time after surgery, in-house vs. external rehabilitation and active/passive rehabilitation sessions on RTS pass rates (*α*=0.05).

**Results:**

Of 104 patients, 82 (45 male, 37 female; mean age 34 ± 11.2 years) completed RTS testing at 27.6 (±4.2) weeks after surgery. Patients completed 30 ± 16 strength and functional training sessions. Mean LSI was 92.8 (±11.1%) for OLH and 88.8 (±16.9%) for SH; mean H/Q was 53.9 (±10.8%) (affected) and 50.2 (±6.8%) (unaffected). Pass rates were 66.7% (OLH), 64.4% (SH), 32.4% (TT), 37.2% (ISO Ext), and 44.2% (ISO Flex). No consistent influencing factors were identified (*p* > 0.05). Fewer active physiotherapy sessions were associated with higher pass rates for SH (OR 0.934; *p* = 0.041) and OLH (OR 0.906; *p* = 0.034).

**Conclusion:**

Less than half of patients met the commonly used RTS criteria at 6.5 months after ACLR, even after isolated ACL injury treated by a single surgeon using a standardized surgical technique and standardized rehabilitation protocol. These findings argue against time-based RTS clearance and support continued criterion-based rehabilitation and RTS testing.

## Introduction

Anterior cruciate ligament (ACL) tears are common knee ligament injuries in young active patients. For a return to unrestricted sports and to prevent an early osteoarthritis patients are commonly advised to undergo an ACL–reconstruction (ACLR) (1, 2). To often, the primary goal of the patients is the earliest possible return to sports (RTS).

Prior surgery, approximately 90% of patients expect a return to their preinjury sports (3). However, 82% of patients return to any kind of sports, 63% return to preinjury sports, 55% to competitive sports level (4). Moreover, re-injury rates after ACLR for young and high-level athletes particularly still remain high (5).

The way back to sports is challenging and a long-lasting process of rehabilitation which needs a consistent guiding and comprehensive monitoring from the first day after surgery. While treatment particularly considering swelling, pain relief and enhancing the range of motion is of major concern within the first weeks, in the following period restoration of lower limb strength and neuromuscular training should be focused. For the RTS management the objective assessment of the functional recovery process is essential for lowered ACL reinjury risk. Assessment commonly consists of of isokinetic strength testing of the knee extensor and flexor muscle and functional test such as tapping agility test (TT) and different variations of hop tests (6–8). In consequence, in the last two decades an alternating trend from an intentionally early RTS 6 months mostly based on time-based RTS consideration towards a criterion-based decision with a delay of more than 6 months after ACLR (9–14).

However, a comparison between different studies due to many different factors is hardly possible. Age, sex, surgical technique, graft type and sports activity level that have been identified as predictive for a successful RTS as well as ACL re-injury (15–20). Of note, either graft type and the surgical technique have been found to impact the subsequent outcome (15, 17, 18, 20). Further co-existing pathologies, such as an osteochondral defect or a meniscus tear, have been found to be negatively predictive to the overall RTS probability (16, 21). In recent studies the rehabilitation process itself has been shown to be predictive for the functional assessment. Examplary, Edwards et al. (22) have reported from 113 following RTS assessement approximately 12 months after ACLR. Patients who completed 6 months of rehabilitation including hop tasks revealed higher RTS pass rate than those who completed less time of rehabilitation.

Numerous studies regarding “RTS after ACLR” have emerged over the past decade and there is still no consensus for standardized RTS management. A major reason for the lacking of RTS consensus might be the various study settings including co-morbidities (e.g., menical tears), different surgical techniques (e.g., hamstring tendon and bone-patellar-bone reconstruction) as well as patient characteristics (e.g., exclusively focused on high-performance athletes). The purpose of the present study was to investigate different patient related factors associated with objective RTS testing after an isolated ACLR following an optimised and standardized outpatient clinic setting.

## Methods

### Patient cohort

For a retrospective study all patients who underwent an ACLR were conducted. Patients aged higher than 60 years or presenting with co-morbidities regarding the cardiopulmonary system, neurological or neuromuscular diseases were excluded from the study. The study was approved by the ethical committee of the Charité University hospital Berlin (EA 1/101/22).

### Surgery and rehabilitation

Surgery was performed by a single surgeon using the ipsilateral hamstring tendon with single bundle technique. Post-surgical care included clinical assessment of wound swelling, range-of-motion (ROM) and initiation of physiotherapy on postoperative day 1. Physiotherapy consisted of manual therapy, lymphatic drainage and soft-tissue techniques to reduce pain and swelling facilitating ROM and early gait rehabilitiation. Based on their preference, patients were subsequently assigned to either the in-house rehabilitation group or the external rehabilitation group. Patients were advised to use limited weight-bearing of 20 kg with crutches and orthosis with limited flexion at 90° for 2 weeks. Full ROM was permitted after 3 weeks. Full weight-bearing without orthosis was allowed 6 weeks post-surgery.

Once joint effusion or pain has been resolved and full ROM has been restored, treadmill gait training, functional exercises as well as concentric and eccentric strengthening of the knee extensors and flexors in a closed kinetic chain were initiated from postoperative week 8–10. Exercises were primarily performed using the leg press or in upright stance, targeting both unilateral and bilateral loading (8, 23). From postoperative weeks 10–12, progressive bilateral strength training was implemented with loading gradually increased up to full body weight. In order to prepare for the demands of running and plyometric activities (8, 24). Between weeks 12 and 16, a structured return-to-running protocol was introduced, beginning with interval sessions alternating 2 min of walking and 2 min of running targeting 10 min of continuous low-intensity running (8). Concurrently, strengthening of the lumbopelvic-hip complex was intensified. Depending on individual strength and neuromuscular control, coordination drills (e.g., running technique exercises using an agility ladder) and basic plyometric exercises, including single-leg hops, were introduced (25, 26). From week 16, low-intensity running on firm outdoor surfaces was permitted, open kinetic chain strengthening exercises were progressively integrated, and sport-specific training was gradually introduced. Throughout all stages, progression was based on individual functional status, quality of movement, and absence of pain. Chronological timelines were used as general guidance only and did not mandate progression between rehabilitation phases (8, 23, 26).

The timing of the RTS assessment was determined by the responsible orthopedic surgeon in coordination with the treating physiotherapists and rehabilitation trainers. The following minimum criteria were required to be met prior to testing: a minimum of 24 weeks post-surgery, absence of pain and joint effusion during and after training, full ROM (full extension and a minimum 130° knee flexion) and the patient's subjective readiness to RTS activities (8, 23, 27).

### Return to sport assessment

Before testing, patients completed a 10-minute warm-up on a the stationary bicycle ergometer or cross-trainer. Isokinetic knee extensor and flexor strength was assessed using a Biodex System 3 dynamometer (Biodex Medical System 3, Inc, Shirley, NY, Figure 1). Both limbs were tested at 60°/s, 180°/s, and 300°/s using 5, 10, and 10 maximal concentric repetitions, respectively; the unaffected limb was always tested first was used to assess the isokinetic strength of knee extensor (ISO Ext) and flexor (ISO Flex) muscles. The hamstring-to-quadriceps muscle (H/Q) ratio was calculated as ipsilateral hamstring peak torque divided by ipsilateral quadriceps peak torque and was evaluated separately from the LSI-based criteria. A Cut off ≥ 50% was used for the H/Q ratio in this heterogenous clinical cohort, consistent with the threshold applied in the study protocol (52–54).

**Figure 1:**
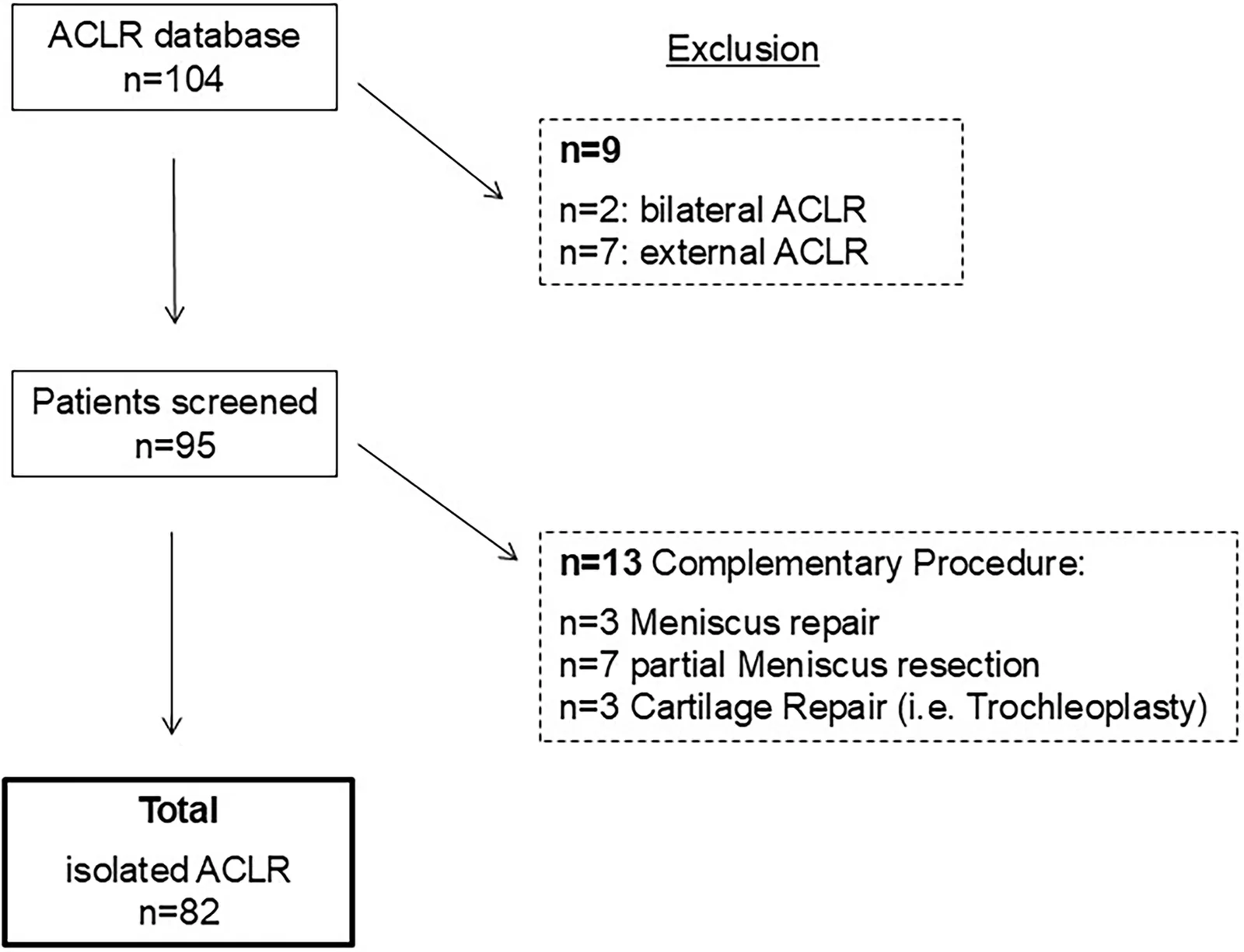
Flowchart of patient recruitment, evaluation and exclusion in the study.

For the isokinetic quadriceps and hamstring strength measures, as well as for the One Leg Hop test (OLH) and the Side Hop test (SH), the LSI was calculated as the affected limb value divided by the unaffected limb value  × 100. An LSI ≥90% was considered the pass criterion for isokinetic strength and hop performance, respectively (8, 11, 23, 28–31).

The OLH and the SH (Figure 2) assessed functional hop performance (29, 32, 33) with a 1-minute rest interval between hop tests. For the OLH, patients hopped on one leg for maximal horizontal distance and were required to demonstrate a stable, balanced landing. Distance was recorded in centimeters (32). For the SH, patients performed side-to-side hops between two floor marks 30 cm apart from each other. The time required to complete 10 valid hops was recorded in seconds. Each leg was tested separately. Touching or crossing the marks invalidated the trial.

**Figure 2:**
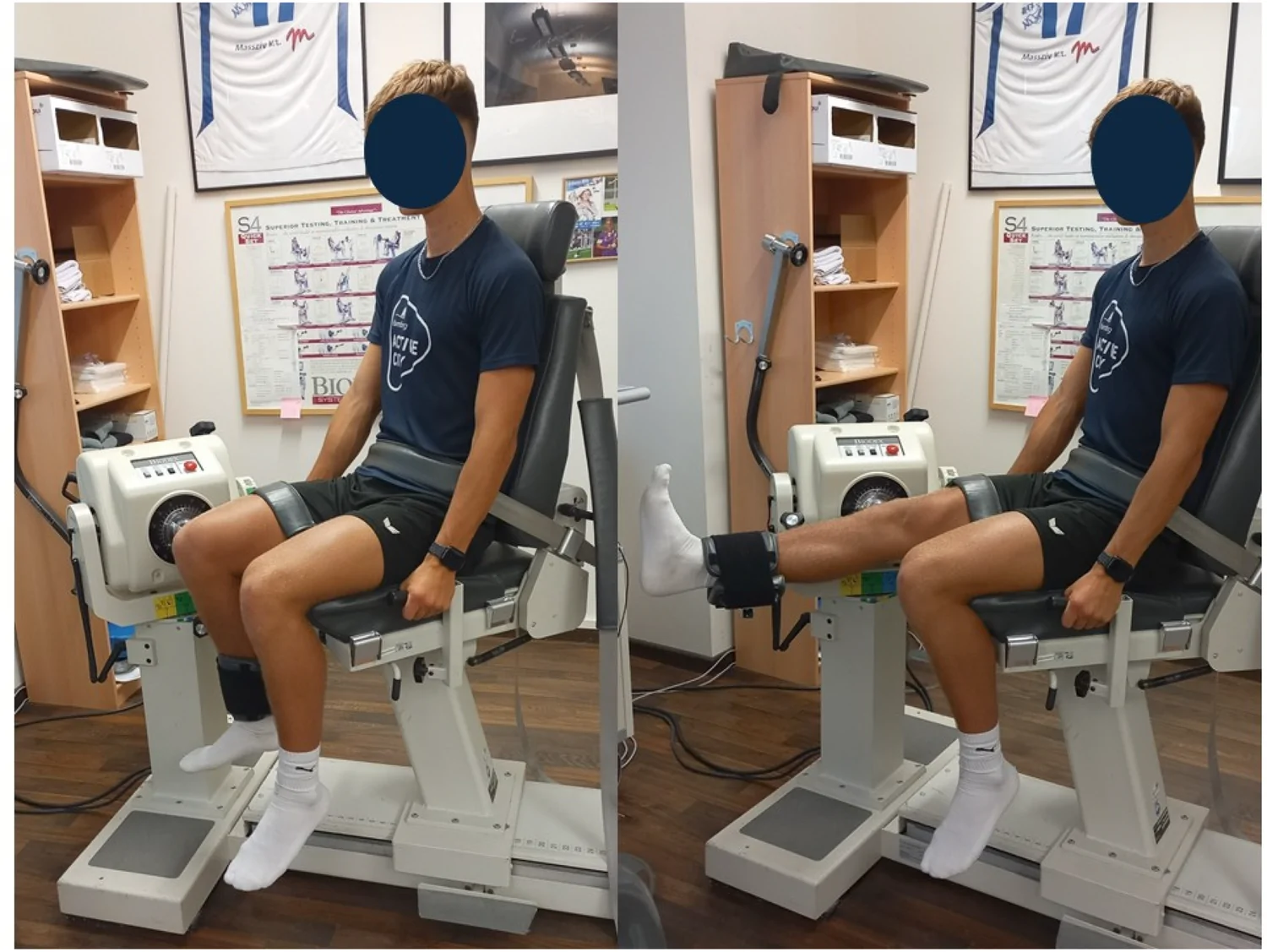
Isokinetic strength testing of the knee extensors and flexors using a Biodex System 3 dynamometer.

The Tapping Agility Test (TT) was used to assess the rapid lower-extremity functional performance. Patients alternately lifted and tapped their feet as quickly as possible for 15 s. The best test result out of two trials was recorded (55). Tapping frequency and ground-contact time were combined into a Tapping Coefficient. In accordance to Voss et al. (34) performance was classified as “poor” for coefficients between 8.0 and 12.0, “moderate” for values between 12.0 and 15.0, “good” for values between 15.0 and 17.5, “very good” for values between 17.5 and 20.0, and “excellent” for values of 20.0 or higher.

### Statistical analysis

SPSS 24.0 (IBM Corp., Armonk, United States) was used for statistical analysis. Continous variables are presented as mean ± standard deviation (SD) for parametric data and as median with range for non-parametric data. Potential differences in rehabilitation time after surgery regarding the rehabilitation setting (in-house vs. external rehabilitation) were tested by use of the Mann–Whitney U test.

The Spearman rank correlation coefficient was used to assess associations between patient-related variables (age, body mass Index (BMI), time after surgery) and the RTS pass rate. Values range from −1 to +1, with values closer to zero indicating weaker and values closer to ±1 indicating stronger associations. Data was interpreted as “no” correlation when 0.0, “weak” when 0.2, “moderate” when 0.5, “strong” when 0.8, and “perfect” when 1.00 (34, 35).

A subgroup analysis compared RTS outcome between patients who completed rehabilitation in-house and those who completed rehabilitation externally.

Nominal logistic regression was used to identify influencing factors on the RTS assessment pass rate. Factors included number of passive and active physiotherapy sessions, sports level and discipline, age, BMI, sex, time after surgery, and the rehabilitation setting. Statistical significance was set at *α*=0.05.

## Results

Between 2015 and 2019 a total of 104 ACLR patients could be identified, from whom 82 cases could be inlcuded in the follow-up analysis. Patients with concomitant pathologies requiring additional procedures e.g., meniscal repair or cartilage defects, other ligamentous injuries as well as patients with bilateral ACLR or external primary ACL surgery, were excluded (Figure 3). Patients completed a mean of 20 (±13.5) sessions of manual therapy and lymphatic drainage and 30 (±16) sessions of strength and functional training. The cohort comprised 14 professional and 68 recreational athletes; 36 ACLR were performed on the left knee and 46 on the right knee. No postoperative complications were recorded and all patients were able to return to their previous sport. Forty-seven patients (57%) completed rehabilitation in-house and 35 (43%) completed rehabilitation elsewhere (Table 1).

**Figure 3:**
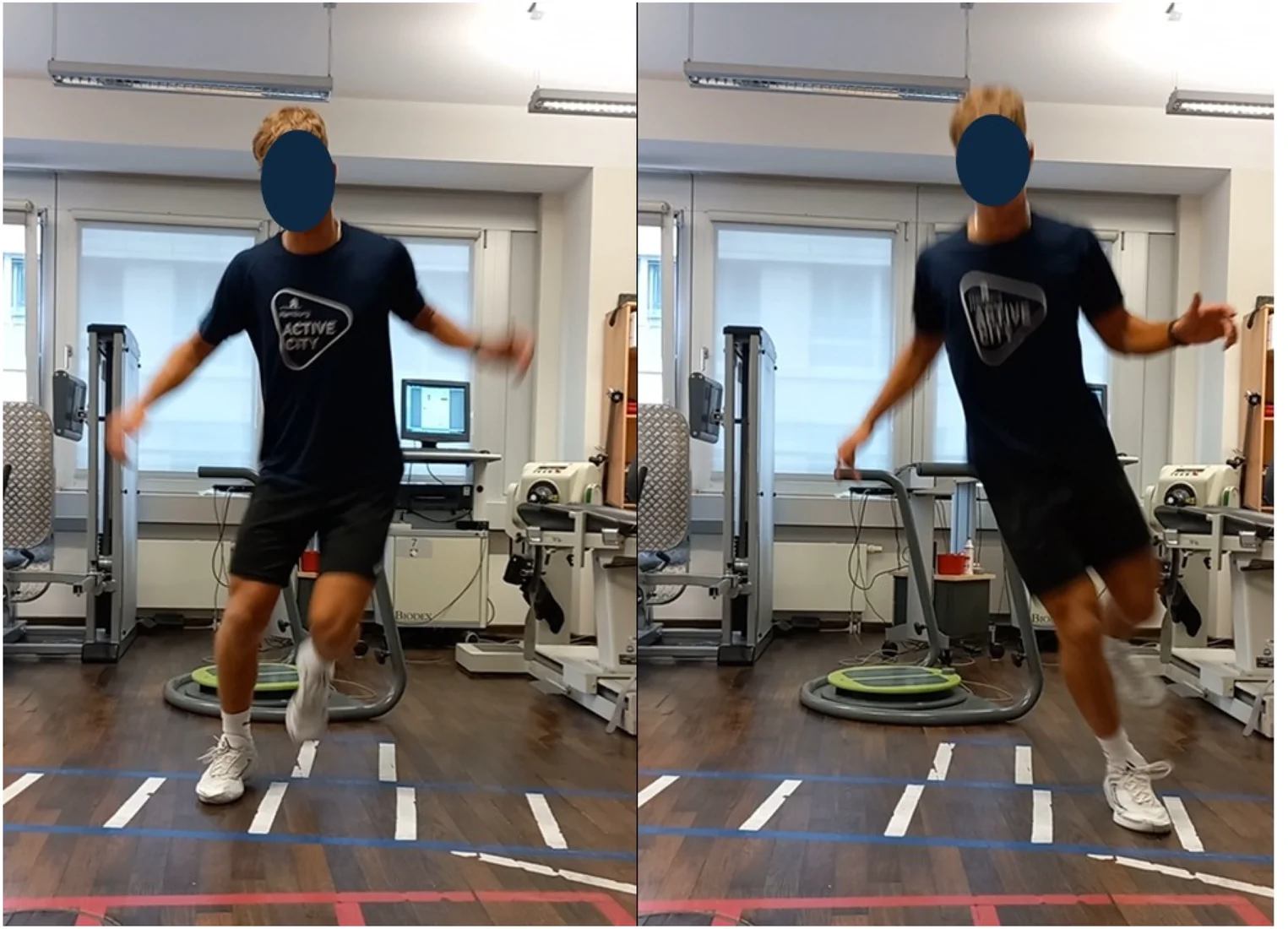
Side hop test (right leg).

**Table 1:** Patient-related variables (*n* = 82).

Variables	Parameter
Age (years)	34 (±11.2)
Sex (female/male)	37/45
BMI (kg·m⁻^2^)	23.4 (±3.2)
Sports level—recreational	68
Sports level—professional	14
Affected side—left/right	36/46
Rehabilitation (in-house/external)	47/35
Physiotherapy (manual therapy, lymph drainage)	20 (±13.5)^a^
Training therapy	30 (±15.8)^a^
Time to RTS assessment (weeks)	27.6 (±4.2)

Values are mean ± SD.

a Total completed sessions.

### RTS assessment

Mean LSI values were 92.8% (±11.1%) for the OLH and 88.8% (±16.9%) for the SH. Mean isokinetic values on the affected side were 84.9% (±16.9%) the ISO Ext and 87.8% (±11.3%) for the ISO Flex (Table 2). Mean ipsilateral H/Q ratio was 53.9% (±10.8%) on the affected side and 50.2% (±6.8%) on the unaffected side (Table 2). LSI ≥90% was achieved by less than half of patients concerning isokinetic strength criteria (ISO Ext: 37.2%; ISO Flex: 44.2%). Pass rate of TT was 32.4%, whereas two thirds of patients passed the hop criteria (OLH: 66.7%; SH: 64.4%).

**Table 2:** Return to sport assessment.

Test	*n*	Affected	Unaffected	Pass criterion	Limb symmetry (%)	Pass rate (%)
ISO extensor DMM/kg %	78	226 (±52.4)	268 DMM/kg % (±57.4)	LSI ≥90%	84.9 (±16.9)	37.2
ISO flexor DMM/kg %	77	120 (±28.3)	134 (±25.0)	LSI ≥90%	87.8 (±11.3)	44.2
ISO H/Q ratio affected/unaffected	77	–	53.9% (±10.8)/50.2% (±6.81)	H/Q ≥ 50%	–	11.7
One-leg hop test (OLH)	72	150 cm (±33.4)	163 cm (±33.3)	LSI ≥90%	92.8 (±11.1)	66.7
Side hop test (SH)	74	15.8 s (±8.25)	13.7 s (±7.09)	LSI ≥90%	88.8 (±16.9)	64.4
Tapping agility test	74	6.75 (±2.36) coefficient	6.52 (±2.14) coefficient	>11.9 coefficient	–	32.4

Values are mean ± SD and absolute/percentage values as applicable.

H/Q, hamstring-to-quadriceps ratio; DMM/kg %, normalized peak torque; LSI, limb symmetry index.

No statistically significant rehabilitation time difference was found for the rehabilitation setting (*p* > 0.05). Correlation analyses showed mostly weak to moderate association regarding the factors a ge, BMI, time after surgery and the RTS outcomes (Table 3). A slight inverse influence of active physiotherapy sessions on the SH (OR 0.934, 95% CI 0.874–0.997; *p* = 0.041) and the OLH (OR 0.906, 95% CI 0.827–0.993; *p* = 0.034) pass rate of the affected side could be detected. No further influencing factors on the achieved test pass rates were found (*p* > 0.05).

**Table 3:** Spearman rank-order correlations between return-to-sport (RTS) tests, patient-related variables.

Test	Age				Body-mass index				Time after surgery (weeks)			
	*N*	Spearman *ρ*	95% CI	*P* - Value	*N*	Spearman *ρ*	95% CI	*P* - Value	*N*	Spearman *ρ*	95% CI	*P* - Value
OLH (unaffected side)	72	−0.339	(−0.529; −0.116)	0.004	72	−0.149	(−0.368; 0.085)	0.211	72	−0.324	(−0.517; −0.100)	0.005
OLH (affected side)	72	−0.320	(−0.514; −0.095)	0.006	72	−0.108	(−0.331; 0.127)	0.369	72	−0.278	(−0.479; −0.050)	0.018
ISO Ext DMM/kg % (unaffected side)	78	0.364	(0.154; 0.543)	0.001	78	−0.223	(−0.400; −0.029)	0.088	78	−0.223	(−0.342; −0.096)	0.260
ISO Ext DMM/kg % (affected side)	78	−0.272	(−0.466; −0.053)	0.016	78	−0.222	(−0.424; −0.000)	0.050	78	−0.222	(−0.317; −0.123)	0.374
ISO Flex DMM/kg % (unaffected side)	77	−0.096	(−0.313; 0.131)	0.407	77	−0.265	(−0.462; −0.044)	0.020	77	−0.224	(−0.405; −0.026)	0.083
ISO Flex DMM/kg % (affected side)	77	0.194	(−0.031; 0.401)	0.090	77	−0.271	(−0.467; −0.050)	0.017	77	−0.230	(−0.432; −0.007)	0.043
ISO H/Q Ratio (unaffected side)	77	0.124	(−0.103; 0.339)	0.283	77	−0.057	(−0.277; 0.169)	0.622	77	−0.105	(−0.321; 0.122)	0.366
ISO H/Q Ratio (affected side)	77	0.036	(−0.190; 0.258)	0.754	77	−0.015	(−0.238; 0.210)	0.897	77	−0.131	(−0.345; 0.096)	0.257
Side Hop (unaffected side)	30	−0.054	(−0.406; 0.312)	0.778	74	−0.229	(−0.618; 0.251)	<.001^a^	74	−0.229	(−0.236; −0.221)	0.948
Side Hop (affected side)	73	0.162	(−0.071; 0.378)	0.172	73	−0.230	(−0.546; 0.143)	0.002	73	−0.080	(−0.304; 0.153)	0.504

Spearman's *ρ*, the corresponding 95% confidence interval (CI), *p*-value, and number of observations (*N*) are reported for each analysis.

a Statistical significance between Side hop (unaffected) and Body-Mass Index (*p* < .001).

## Discussion

Main findings of the present study were that less than half of the patients met the targeted RTS criteria approximately 6.5 months after ACLR in an optimized therapeutic setting. It incorporated a single surgeon, a standardized hamstring autograft technique, and a standardized rehabilitation protocol including RTS assessment supervised by the responsible surgeon. The results therefore indicate that functional recovery is frequently incomplete at 6.5 months after surgery, even under standardized conditions and in absence of concomittent injuries.

Our results match those of recently reported data (6, 30, 36–39) strongly indicating that recovery is incomplete at common reported RTS time points. Krych et al. (40) reported from 224 patients who underwent an ACLR with 23% (52/224) RTS test pass 6 months after surgery. Moreover, Kyritsis et al. (6) reported a pass rate of 29% at 6 months and of 52% at 12 months among 158 male professional athletes. The comparatively higher pass rate of patients in the present study may be the result of the selected inclusion criteria inlcuding isolated ACL injuries without cocomittant injuries. However, also in our study a large proportion of patients did not meet the RTS criteria at the expected time point of the involved surgeon.

Clearing an athlete for RTS is complex and must be understood as a decision-making process incorporating multiple factors (41). Although consensus of the validity and the optimal composition of individual RTS assessment remains lacking, objective measures of the functional performance provide clinical relevant information that cannot be captured by time-based criteria (13).

The RTS assessment remains inconsistently implemented in clinical practice (32). Barber-Westin et al. (32) reported its implementation only in a minority of studies, whereas many studies relied solely on time elapsed from surgery. This is of relevant concern because failure in meeting the RTS criteria is associated with high risk of a secondary ACL injury [Kyritsis, 2016 #31], particularly among young patients and athletes (42). The present data supports a criterion-based RTS progression rather than time-based clearance. However, relevant influenceing factors could not be identified. RTS testing should therefore be used as a longitudinal monitoring tool as well as a decision-making instrument (43, 44). This involvea all stakeholders, the surgeon, the physiotherapists or rehabilitation trainers and the patient (48, 56, 57).

An important and somewhat unexpected finding was the absence of a significant difference in RTS outcomes between the 47 patients (57%) who completed rehabilitation in-house and the 35 patients (43%) who completed rehabilitation elsewhere. The in-house rehabilitation provided closer access to the surgeon and standardized rehabilitation within the same clinical environment, yet it did not result in superior RTS test outcomes. Several explanations are possible, including differences in patient adherence, training frequency and intensity, progression of external loads, fear of overloading the graft, and the heterogeneous sport demands of this real-world cohort. This raises the important consideration that adequate rehabilitation requires more than simply providing patients with a standardized postoperative rehabilitation protocol.

## Limitations

This study has several limitations that should be acknowledged. First, in the external rehabilitation group, it could not be ensured that patients underwent rehabilitation in accordance with the standardized postoperative protocol. As a result, it remains unclear whether adherence to the postoperative rehabilitation protocol may have influenced RTS test outcomes during the recovery phase. This limitation reflects a fundamental drawback of the retrospective study design, in which control over standardized follow-up and intervention is limited.

Nevertheless, the analysis beween in-house and external rehabilitation revealed no significant differences in RTS outcomes, suggesting that the influence of rehabilitation setting was less incisive in this cohort. However, this finding should be interpreted cautiously, given the lack of detailed rehabilitation data in the external group. Particularly in community-based care, RTS rehabilitation is often unsupervised and largely dependent on patient adherence. Despite widely available evidence-based protocols, their implementation in real-world settings is inconsistent, resulting in inadequate supervision, particularly beyond 3 months (8, 45–47).

The RTS test battery used commonly accepted measures such as isokinetic strength testing and single-leg hop tests, evaluating the performance via the LSI ≥90%. Traditionally, an LSI between ≥ 85% to 90% were interpreted as appropriate for RTS (29, 48, 49) while it is still not clarified whether small side-to-side differences affect the RTS safety or not. Other authors considered an LSI higher than 100% for high demanding sports (e.g., pivoting and cutting) being a safe cut-off value (50). While these metrics are widely applied in clinical and research settings, the LSI has been criticized for its limited ability to capture qualitative movement patterns. LSI-based assessments may potentially assess asymmetries in movement quality and neuromuscular control. It is therefore recommended to include alternative qualitative assessment tools such as the Drop Jump Test and the Landing Error Scoring System (LESS) (51).

## Conclusion

Our findings demonstrate that less than half of patients meet commonly accepted RTS criteria at 6.5 months post ACLR, even in the reported cohort of cases with isolated ACL injuries treated by a single surgeon, a standardized surgical technique and rehabilitation protocol. This indicates that functional recovery is frequently incomplete at commonly used RTS time points. Time after surgery alone is therefore considered as an insufficient parameter to determine readiness to return to sport. Neither age, BMI or time after surgery, nor the number of documented rehabilitation sessions reliably identified patients readiness for return to sport. Our results highlight that the subjective impression of the surgeon regarding RTS readiness should not replace objective criterion-based testing. Instead, structured RTS testing should be conducted more regularly throughout the rehabilitation process following ACLR to guide targeted rehabitliation and improve functional recovery.

## Data Availability

The raw data supporting the conclusions of this article will be made available by the authors, without undue reservation.
